# Quantification and Application of Potential Epigenetic Markers in Maternal Plasma of Pregnancies with Hypertensive Disorders

**DOI:** 10.3390/ijms161226201

**Published:** 2015-12-15

**Authors:** Hyun Jin Kim, Shin Young Kim, Ji Hyae Lim, Dong Wook Kwak, So Yeon Park, Hyun Mee Ryu

**Affiliations:** 1 Laboratory of Medical Genetics, Medical Research Institute, Cheil General Hospital and Women’s Healthcare Center, Seoul 100-380, Korea; bluemint1999@hanmail.net (H.J.K.); chlorella@empal.com (S.Y.K.); roollala@hanmail.net (J.H.L.); paranip5@hanmail.net (S.Y.P.); 2 Department of Obstetrics and Gynecology, Cheil General Hospital and Women’s Healthcare Center, Dankook University College of Medicine, Seoul 100-380, Korea; drsweety@naver.com

**Keywords:** epigenetic marker, cell-free fetal DNA, cell-free total DNA, pregnancies with hypertensive disorders

## Abstract

The aim of this study was to evaluate quantitative aberrations of novel fetal-specific epigenetic markers in maternal plasma of pregnancies with hypertensive disorders. We compared the concentrations of *DSCR3, RASSF1A,* and *SRY* as cell-free fetal DNA markers in 188 normal pregnancies, 16 pregnancies with early-onset preeclampsia (EO-PE), 47 pregnancies with late-onset preeclampsia (LO-PE), and 29 pregnancies with gestational hypertension (GH). The concentrations of all markers were significantly correlated with gestational age (*p* < 0.001 for all). Strong positive correlations were also observed between *DSCR3* and *SRY* (*r* = 0.471, *p* < 0.001), as well as between *RASSF1A* and *SRY* (*r* = 0.326, *p* = 0.015) and between *DSCR3* and *RASSF1A* (*r* = 0.673, *p* < 0.001). The concentrations of *DSCR3* and *RASSF1A* in the EO-PE were significantly higher at 24–32 weeks and onwards (*p* < 0.05 for both). In the LO-PE, *DSCR3* and *RASSF1A* concentrations were significantly higher only at 33–41 weeks compared with the controls. The concentrations of all markers in the GH group were not significantly different from those in the control group. This study is the first demonstration that *DSCR3* is a novel epigenetic marker that can be an alternative to the *RASSF1A* for the prediction of EO-PE.

## 1. Introduction

Hypertensive disorders in pregnancy are a leading cause of maternal, fetal, and neonatal morbidity and mortality. Hypertension represents the most common medical complication of pregnancy, affecting 10%–22% of pregnancies, and has been classified into four conditions, reflecting potential differences in etiology and pregnancy outcomes: namely gestational hypertension (GH), chronic hypertension (CH), preeclampsia (PE) and preeclampsia superimposed on chronic hypertension [1,2]. Hypertensive pregnancies can result in maternal cerebral hemorrhage, disseminated intravascular coagulation, hepatic failure, acute renal failure, pulmonary edema, placental abruption, the fetus being at increased risk of intrauterine growth restriction (IUGR), intrauterine death, and preterm births. In the most serious cases of hypertensive disorders in pregnancy, the mother develops PE, which can threaten the lives of both the mother and the fetus [1,2]. Therefore, the need for a clinically applicable test that can identify women at risk of developing PE before the onset of symptoms is warranted.

The finding of circulating cell-free fetal DNA (cffDNA) in maternal plasma by Lo *et al.* in 1997 opened up new perspectives in noninvasive prenatal diagnosis of pregnancy complications [3]. cffDNA in maternal plasma is of placental origin shed from the syncytiotrophoblast as apoptotic fragments due to normal cell turnover, and is released into the maternal circulation. During the past decade, several reports have described associations between cffDNA and pregnancy complications. These results have been successfully applied for the determination of fetal sex, RhD status, fetal aneuploidies, and monitoring of pregnancy complications associated with placental dysfunction such as PE and IUGR [4,5,6,7,8].

Recently, epigenetic differences between maternal and fetal DNA have been explored as a universal fetal-specific marker, independent of fetal sex and genetic polymorphism. The promoter of the *RASSF1A* gene is hypermethylated in term placenta, whereas hypomethylated in maternal blood cells.

Tissue-specific DNA methylation patterns makes it possible to distinguish fetal DNA from the background maternal DNA [9]. For this reason, several studies quantifying cffDNA in maternal plasma in pregnancies with PE and IUGR have usually tested the *RASSF1A* gene as an epigenetic marker for predicting placental dysfunction [10,11,12]. However, there is controversy over the potential use of the *RASSF1A* gene in maternal plasma as a marker of PE, and evidence for other epigenetic markers for the early prediction of pregnancy complications including PE is insufficient.

Some studies have investigated gene expression profiles in human placentas with PE using microarrays to identify novel biomarkers [13,14,15,16]. However, none of these studies investigated candidate genes for detection in maternal plasma, and their potential as clinical markers has not been examined thoroughly. In a previous study, we analyzed human chromosome 21 with a high**-**resolution tiling microarray using a methyl**-**CpG binding domain-based protein (MBD) method to find Down-specific characteristics of fetal epigenetic markers [17]. Therefore, the aim of the present study was to identify differentially methylated regions that are specifically hypermethylated in placenta and hypomethylated in maternal blood cells, based on previous methylation profiling data, and evaluated quantitative aberrations of candidate genes as potential epigenetic markers in maternal plasma in pregnancies with hypertensive disorders throughout pregnancy.

## 2. Results and Discussion

### 2.1. Results

#### 2.1.1. Clinical Characteristics

The clinical characteristics of each of the study groups (controls, EO-PE, LO-PE and GH) are presented in Table 1. There were no significant differences in maternal age, nulliparity, gestational age at sampling, sex ratio of fetuses, alcohol intake, and tobacco use among the groups (*p* > 0.05 for all). Body mass index and blood pressure were higher (*p* < 0.05 for all), while gestational age at delivery and birth weight were significantly lower in all patient groups than the control group (*p* < 0.05 for all).

**Table 1:** Clinical characteristics of the subjects.

Characteristics	Controls (*n* = 188)	EO-PE (*n* = 16)	LO-PE (*n* = 47)	GH (*n* = 29)	*P* ^a^	*P* ^b^	*P* ^c^	*P* ^d^	*P* ^e^	*P* ^f^
Maternal age (years)	33.0 (31.0–36.0)	34.0 (31.6–36.0)	34.1 (33.0–36.1)	35.0 (33.0–37.0)	0.204	0.061	0.059	0.383	0.393	0.809
BMI (kg/m^2^)	20.2 (18.8–21.9)	22.3 (22.1–29.4)	22.6 (20.3–23.5)	22.8 (21.2–30.5)	0.000	0.000	0.000	0.073	0.948	0.044
Nulliparity	76 (40.4)	6 (37.5)	24 (51.1)	16 (55.2)	0.819	0.187	0.135	0.397	0.353	0.815
Alcohol intake	44 (23.4)	6 (37.5)	13 (27.7)	5 (17.2)	0.229	0.543	0.460	0.534	0.161	0.408
Tobacco use	2 (1.1)	–	–	–	–	–	–	–	–	–
Maximum SBP (mmHg)	109.0 (101.0–118.0)	162.0 (160.0–173.0)	147.0 (140.5–150.5)	157.0 (150.5–167.0)	0.000	0.000	0.000	0.000	0.387	0.000
Maximum DBP (mmHg)	63.0 (57.0–69.0)	99.0 (93.0–100.0)	90.0 (86.0–94.0)	96.0 (89.0–98.5)	0.000	0.000	0.000	0.035	0.355	0.077
Birthweight (g)	3290.0 (3040.0–3495.0)	2265.0 (1878.8–2362.5)	2920.0 (2572.5–3297.5)	3012.5 (2800.0–3290.0)	0.000	0.000	0.001	0.002	0.000	0.384
Sex ratio of fetus (male:female)	92:96	10:6	16:31	11:18	0.298	0.067	0.269	0.076	0.133	0.807
GA at delivery (weeks)	39.5 (39.1–40.3)	35.8 (34.4–36.4)	38.3 (36.8–39.3)	38.6 (38.2–39.5)	0.000	0.000	0.006	0.003	0.000	0.032
GA at sampling (weeks)	–	–	–	–	–	–	–	–	–	–
First trimester	12.1 (8.4–12.4)	12.0 (11.1–12.3)	12.3 (11.7–12.5)	12.3 (12.2–12.3)	0.850	0.382	0.205	0.571	0.567	0.970
Sceond trimester	21.2 (16.4–25.0)	20.9 (16.6–25.2)	18.0 (16.6–24.0)	17.9 (16.3–24.4)	0.722	0.060	0.090	0.451	0.189	0.903
Third trimester	36.5 (36.1–37.0)	36.3 (35.3–36.6)	36.9 (36.2–38.2)	36.6 (36.6–37.7)	0.063	0.329	0.100	0.187	0.073	0.967

BMI, body mass index; SBP, systolic blood pressure; DBP, diastolic blood pressure; GA, gestational age; EO-PE, early-onset preeclampsia; LO-PE, late-onset preeclampsia; GH, gestational hypertension. Data are presented as median (IQR, interquartile range) or number (%); ^a^ EO-PE *_VS_.* controls; ^b^ LO-PE *_VS_.* controls; ^c^ GH *_VS_*. controls; ^d^ EO-PE *_VS_*. LO-PE; ^e^ EO-PE *_VS_*. GH; ^f^ LO-PE *_VS_*. GH.

#### 2.1.2. Selection of Fetal-Specific Hypermethylated CpG loci by High-resolution Tiling Array Analysis

**Figure 1:**
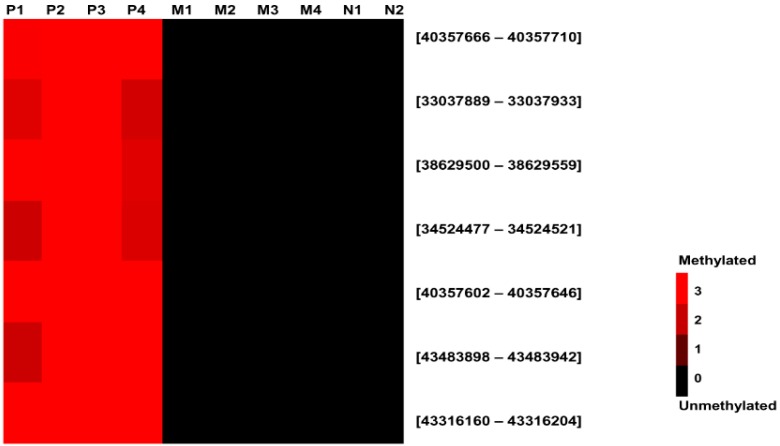
Methylation profiling of 7 identified regions in the subjects using the high-resolution tiling arrays. The red and black colors indicate high expression and non-expression, respectively. P, placenta; M, maternal blood cells; N, non-pregnant women blood cells.

We selected regions that were hypermethylated in placenta and hypomethylated in maternal blood cells as potential fetal DNA markers. Selection criteria were 1) differential log_2_ ratio cut off greater than 2.0 between placental and maternal DNA and 2) a mean baseline (maternal DNA) log_2_ ratio of less than 0.001. Seven regions were identified as differentially methylated in placenta compared with maternal blood cells (Table 2 and Figure 1).

**Table 2:** Methylation levels of selected regions according to tissue type.

Target Regions	Chr.	Description	Probe Position		Placenta (Log_2_)	Normal Blood (Log_2_)	
			Start	Stop		Pregnant	Non-Pregnant
*SOD1*	21	Inside	33037889	33037933	2.62 ± 0.53	0.00 ± 0.00	0.00 ± 0.00
*DSCR3*	21	Inside	38629500	38629559	2.77 ± 0.43	0.00 ± 0.00	0.00 ± 0.00
*C2CD2*	21	Inside	43316160	43316204	3.45 ± 1.02	0.00 ± 0.00	0.00 ± 0.00
*UMODL1*	21	Inside	43483898	43483942	3.14 ± 0.84	0.00 ± 0.00	0.00 ± 0.00
ENST00000433952: -7924	21	Promoter	40357602	40357646	3.39 ± 0.77	0.00 ± 0.00	0.00 ± 0.00
ENST00000433952: -7988	21	Promoter	40357666	40357710	3.52 ± 1.12	0.00 ± 0.00	0.00 ± 0.00
ENST00000450830: -4439	21	Promoter	34524477	34524521	2.85 ± 0.82	0.00 ± 0.00	0.00 ± 0.00

Data are presented as mean ± SE.

#### 2.1.3. Confirmation of High-Resolution Tiling Array Data Using Bisulfite Direct Sequencing

**Figure 2:**
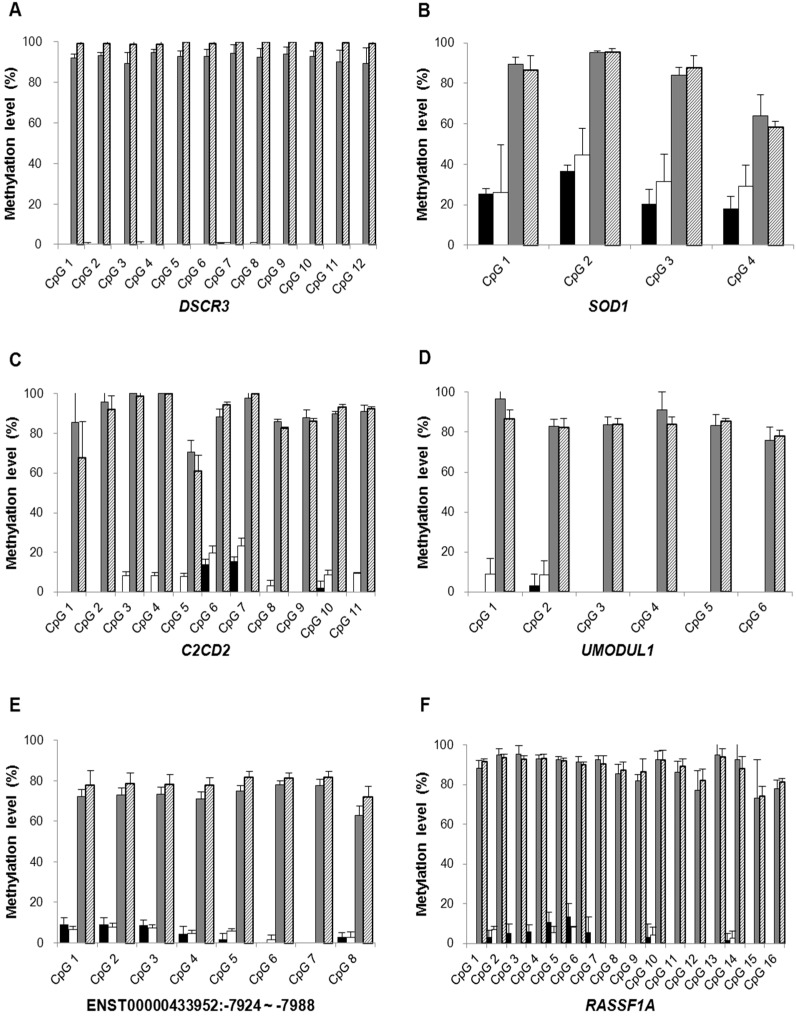
DNA methylation levels of each CpG site in *DSCR3*, *SOD1*, *C2CD2*, *UMODL1* and ENST00000433952:-7924~-7988 regions selected from array data and *RASSF1A* region by bisulfite direct sequencing. The methylation levels each CpG site of these 5 regions and *RASSF1A* region quantified in 8 pairs of maternal blood cells and placenta samples from PE and normal pregnancies: (**A**) *DSCR3*; (**B**) *SOD1*; (**C**) *C2CD2*; (**D**) *UMODL1*; (**E**) ENST00000433952:-7924~-7988; (**F**) *RASSF1A*. Data are presented as mean ± SE. Solid black bars, maternal blood cells from normal pregnancy; open bars, maternal blood cells from PE pregnancy; gray bars, placental tissues from normal pregnancy; stripes bar, placental tissues from PE pregnancy.

We confirmed methylation levels of each CpG site in *DSCR3*, *SOD1*, *C2CD2*, *UMODL1* and ENST00000433952:-7924~-7988 regions, excluding ENST00000450830:-4439 using bisulfite direct sequencing. Consistent with the array data, the methylation levels of each CpG site in five selected regions were significantly increased in placenta compared with maternal blood cells (*p* < 0.001 for all CpG sites in all selected regions). However, the methylation levels were not different between PE and normal placentas (*p* > 0.05 for all CpG sites in all selected regions). Among these selected regions, *DSCR3* was considered as our top priority for developing fetal-specific epigenetic markers in maternal plasma because all CpG sites in *DSCR3* were clearly methylated in placenta compared with maternal blood cells. Moreover, *DSCR3* has the largest number of CpG sites, which facilitated the MBD capture of methylated DNA. In addition, *RASSF1A*, a known cffDNA marker, was confirmed to have all CpG sites hypermethylated in placentas (Figure 2 and Figure S1).

#### 2.1.4. Quantification of DSCR3, RASSF1A, SRY and GADH in Maternal Plasma of Normal Pregnancies

**Figure 3:**
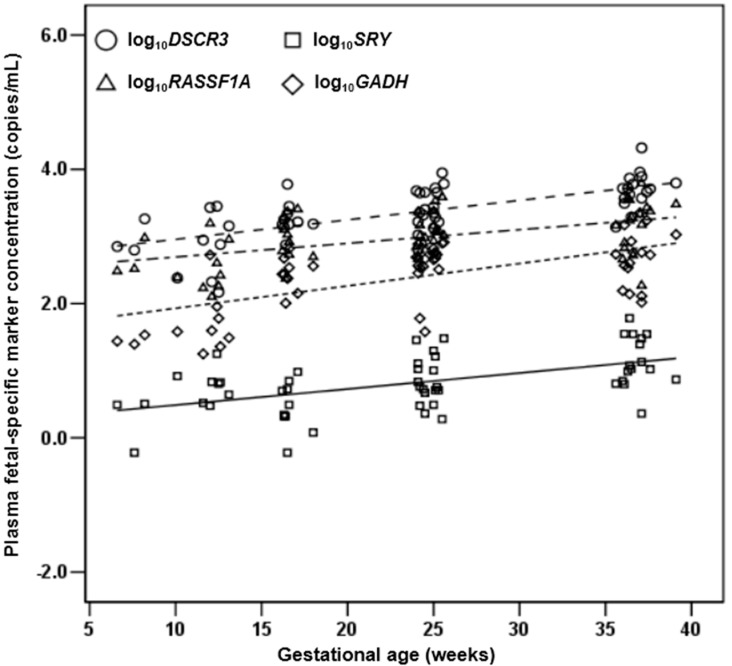
Relationship between concentrations of all markers and gestational age in the control group. The mean value for *DSCR3* is shown as dashes; for *RASSF1A* as dash-dots; for *SRY* as solid; for *GAPDH* as dots. The *Y* axis represents a logarithmic scale.

**Figure 4:**
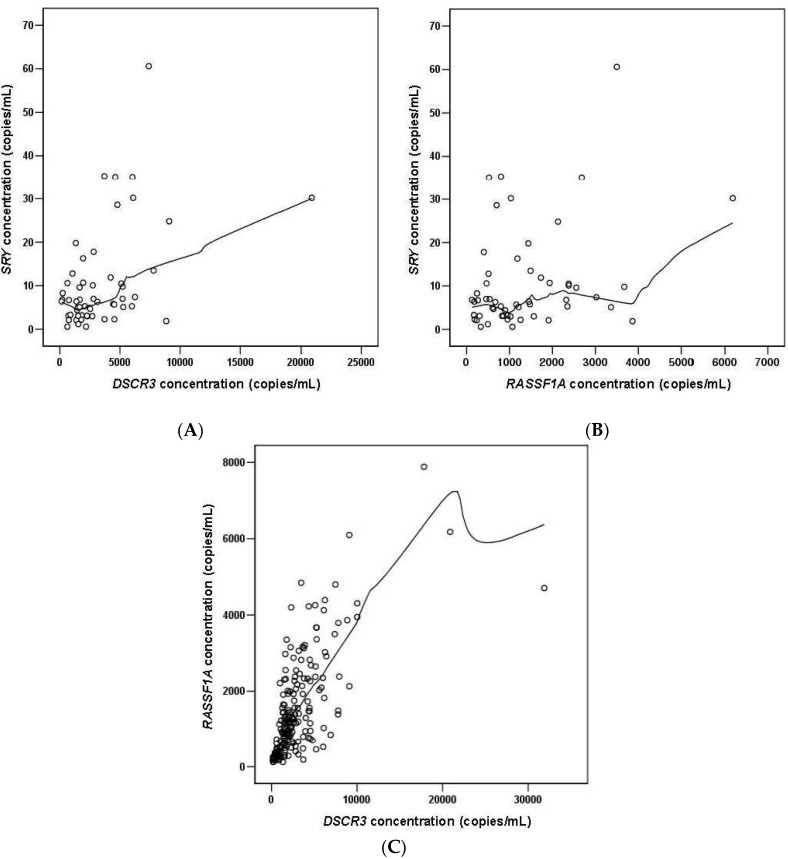
Relationship between (**A**) *DSCR3* and *SRY,* (**B**) *RASSF1A* and *SRY,* (**C**) *DSCR3* and *RASSF1A* concentrations in the control group. The solid black lines represent lines of best fit (estimated with Loess regression).

We investigated the possibility of the detection of cell-free fetal *DSCR3* in maternal plasma, and feasibility of *DSCR3* as a potential epigenetic marker using real time PCR in 188 uncomplicated singleton pregnancies at 6 to 41 weeks of gestation. After MBD capture, *DSCR3* and *RASSF1A* were detectable in maternal plasma of all pregnant women without failure. Both *SRY* and *GAPDH* were detected and amplified in all male-bearing pregnancy pregnancies. The concentrations of all markers were correlated with gestational age at sampling (*DSCR3*: *r* = 0.695; *RASSF1A*: *r* = 0.452; *SRY*: *r* = 0.522; *GAPDH*: *r* = 0.609, *p* < 0.001 for all, Figure 3). As shown in Figure 4, strong positive correlations were observed between *DSCR3* and *SRY* (*r* = 0.471, *p* < 0.001), as well as between *RASSF1A* and *SRY* (*r* = 0.326, *p* = 0.015) and between *DSCR3* and *RASSF1A* (*r* = 0.673, *p* < 0.001).

#### 2.1.5. DSCR3 and RASSF1A Concentrations in Maternal Plasma between Normal Pregnancies and Pregnancies with Hypertensive Disorders

Comparisons of *DSCR3* and *RASSF1A* concentrations in maternal plasma between control and patient groups throughout pregnancy are shown in Table 3. To evaluate increasing points, we analyzed mean concentrations of *DSCR3* and *RASSF1A* in maternal plasma obtained within nine week gestational age intervals from 6 to 41 weeks. At 6 to 23 weeks, *DSCR3* and *RASSF1A* concentrations in all patient groups were not significantly different from the control group. At 24–32 weeks, concentrations of *DSCR3* and *RASSF1A* were significantly higher only in the EO-PE group. At 33–41 weeks, *DSCR3* and *RASSF1A* concentrations were significantly higher in the LO-PE group as well as in the EO-PE group. However, there were no significant differences in *DSCR3* and *RASSF1A* concentrations between GH and control groups throughout pregnancy (*p* > 0.05 for all). Statistically significant differences among patient groups were not observed (*p* > 0.05 for all).

### 2.2. Discussion

In this study, we identified a potential hypermethylated cffDNA (*DSCR3*) epigenetic marker. The concentration of *DSCR3* was correlated with gestational age, and strong positive correlation between cffDNA concentrations were observed when compared to those determined using the *SRY* gene as a specific fetal male marker and *RASSF1A* as a well-known universal epigenetic marker. This result supports the fetal origin of a universal fetal epigenetic marker as well because *SRY* gene in maternal plasma originates from the placenta. Moreover, the concentrations of *DSCR3* and *RASSF1A* were significantly increased in EO-PE before the onset of symptoms, but not in cases with LO-PE and GH.

**Table 3:** Maternal plasma concentrations of epigenetic markers in control and patient groups during pregnancy.

Marker	Controls	EO-PE	LO-PE	GH	*P* ^a^	*P* ^b^	*P* ^c^	*P* ^d^	*P* ^e^	*P* ^f^
6–14 weeks	(*n* = 52)	(*n* = 4)	(*n* = 10)	(*n* = 10)	–	–	–	–	–	–
*DSCR3*	890.9 (538.9–1530.0)	1007.7 (721.5–1454.3)	939.1 (699.6–1718.2)	1098.3 (561.7–2073.6)	0.818	0.467	0.566	0.945	0.733	0.739
*RASSF1A*	445.6 (262.9–936.4)	858.0 (652.6–1215.2)	595.2 (382.1–807.2)	450.1 (385.1–719.7)	0.118	0.503	0.444	0.374	0.188	0.853
15–23 weeks	(*n* = 31)	(*n* = 3)	(*n* = 16)	(*n* = 7)	–	–	–	–	–	–
*DSCR3*	1779.7 (1443.8–2590.6)	2594.0 (2171.5–3075.6)	2573.7 (2159.9–2936.4)	1692.8 (1220.7–2009.1)	0.289	0.121	0.580	0.958	0.183	0.076
*RASSF1A*	1333.8 (727.9–2403.3)	2305.5 (1571.0–2842.2)	1601.4 (1154.4–2036.8)	1013.3 (716.5–1375.0)	0.449	0.946	0.282	0.421	0.183	0.103
24–32 weeks	(*n* = 47)	(*n* = 5)	(*n* = 7)	(*n* = 5)	–	–	–	–	–	–
*DSCR3*	2521.3 (1824.9–4438.2)	6772.5 (5251.1–7876.2)	3295.0 (1118.3–6765.2)	2725.5 (1372.9–6415.1)	0.018	0.920	0.857	0.268	0.222	1.000
*RASSF1A*	1228.3 (827.6–2357.1)	3100.8 (2359.7–4600.0)	819.1 (610.1–1177.3)	1765.5 (406.2–2965.0)	0.032	0.212	1.000	0.149	0.222	0.639
33–41 weeks	(*n* = 58)	(*n* = 4)	(*n* = 14)	(*n* = 7)	–	–	–	–	–	–
*DSCR3*	4212.9 (2841.1–6162.5)	10,632.4 (8426.2–16,027.7)	11,164.3 (7733.5–13,909.9)	6985.4 (5070.0–8881.8)	0.001	0.000	0.079	0.721	0.109	0.056
*RASSF1A*	1777.2 (930.2–2674.1)	4002.8 (3449.6–5142.4)	5051.3 (3441.6–6787.2)	2979.7 (1915.7–3763.8)	0.008	0.000	0.173	0.721	0.109	0.016

EO-PE, early-onset preeclampsia; LO-PE, late-onset preeclampsia; GH, gestational hypertension. Maternal plasma concentrations are shown in copies/mL. Data are given as median (IQR). ^a^ EO-PE *_VS_.* controls; ^b^ LO-PE *_VS_.* controls; ^c^ GH *_VS_.* controls; ^d^ EO-PE *_VS_.* LO-PE; ^e^ EO-PE *_VS_.* GH; ^f^ LO-PE _*VS*_*.* GH.

Several studies have investigated gene expression and methylation profiling in maternal blood cells and placentas with PE using microarrays [13,14,15,16]. However, the suggested candidate genes were not validated by noninvasive detection in maternal plasma and were not evaluated for their potential clinical applications. Although we did not assess DNA methylation changes in placentas with PE by microarray, and were therefore unable to characterize the pathological differences between PE and normal pregnancies, this study provided the alternative epigenetic marker by confirming the presence of *DSCR3* in maternal plasma and evaluating quantitative aberrations of *DSCR3* and *RASSF1A* in maternal plasma in pregnancies with hypertensive disorders. Our results found that *DSCR3* concentrations as well as *RASSF1A* were significantly elevated in both EO-PE and LO-PE compared with the controls. Particularly, *DSCR3* and *RASSF1A* concentrations in the EO-PE group were significantly different from 24 weeks of gestation.

Prior studies have shown increases in concentrations of cffDNA in the plasma of pregnant women before the clinical onset of PE and/or IUGR. Cotter *et al.* reported concentrations of *SRY* in maternal plasma increased at an average 16 weeks’ gestation, and Papantoniou *et al.* revealed differences in the levels of *RASSF1A* at 11–13 weeks’ gestation in women who subsequently developed PE compared with uncomplicated pregnancies [10,18]. In contrast, Crowley *et al.* described no significant differences in *SRY* quantitation before 20 weeks in women with PE or IUGR, and Poon *et al.* also reported no alterations of cffDNA counts by chromosome-selective sequencing in pregnancies with complications [19,20]. Other studies have reported that cffDNA levels were significantly increased in cases of EO-PE or severe PE prior to disease onset, but not in cases with LO-PE or mild PE. Levin *et al.* found a two stage elevation of *DYS1* before the development of PE, one occurring at 17–28 weeks and the other within three weeks of onset only in patients with severe disease [21]. Additionally, Sifakis *et al.* and Illanes *et al.* also demonstrated that *DYS14* concentrations were significantly higher only in cases of EO-PE [22,23]. Likewise, we also found that cffDNA concentrations (*DSCR3* and *RASSF1A*) were significantly different at 24–32 weeks onwards in EO-PE and after 33 weeks of gestation in LO-PE.

These contradictory results might be explained by heterogeneity among studies. Gestational ages of maternal sampling points vary among studies, as did timing of estimation of cffDNA. Moreover, different types of PE such as EO-PE, LO-PE, and any type of PE can result in different study outcomes, since EO-PE and LO-PE may have different pathologic features. EO-PE is suggested to result from failure of proper uterine spiral artery transformation during the early stages of placentation, which induces aberrant placental perfusion with increased apoptosis of trophoblasts [24,25]. Meanwhile, LO-PE is considered to be a maternal syndrome, usually associated with normal placental development and predisposition to maternal hypertension [25,26]. In concordance with these descriptions, our findings of different increasing points of concentrations of all markers according to severity of PE support the hypothesis that increasing levels of cffDNA in maternal plasma are associated with degree of impairment in placental perfusion.

Additionally, our results showed no aberrations in concentrations of any markers in the GH group. This result could be explained by findings by Correa *et al.* and Maloney *et al.* comparing placental pathology in pregnancies with GH and PE [27,28]. They reported that GH and PE had placental pathologic features in common, but placentas from pregnancies with PE were characterized by a higher number of syncytial knots and by higher rates of decidual vasculopathy and villous infarction, suggesting that placental ischemia is confined to PE. Furthermore, Noori *et al.* found that endothelial dysfunction and imbalance between pro- and anti-angiogenic factors are characteristics specific to PE but not GH [29]. Collectively, it has been suggested that GH and PE are two distinct disease entities.

The ideal universal fetal DNA marker should show high homogeneity between individual fetuses and be easy to exclude maternal DNA [30]. However, since abnormal methylation of the *RASSF1A* gene promoter can also be found in patients with certain malignant diseases, it requires special attention to the applications in patients with a known history of cancer. Therefore, for patients with a known history of cancer, alternative cffDNA markers may be necessary [9]. On the other hand, previous studies have been suggested that abnormal concentrations of prenatal screening serum biomarkers in screening for fetal aneuploidy may be associated with adverse pregnancy outcomes such as PE, IUGR, preterm delivery, and fetal loss. And, therefore, several studies have been reported utility of serum biomarkers for screening adverse pregnancy outcomes [31,32,33,34]. Similarly, the *DSCR3* (Down syndrome critical region 3) gene*,* located on chromosome 21 and which contributes significantly to the pathogenesis of many characteristics of Down syndrome, could be a great potential universal epigenetic marker for not only hypertensive disorders but also other pregnancy complications such as Down syndrome.

## 3. Materials and Methods

### 3.1. Study Participants and Samples

This study was approved by the Institutional Review Board and the Ethics Committee of Cheil General Hospital (#CGH-IRB-2013-54), and informed consent was obtained from all study participants. We performed a nested case-control study of women with singleton pregnancies recruited from the Department of Obstetrics and Gynecology at Cheil General Hospital between August 2010 and August 2014. Ten milliliters of maternal blood samples were collected at 6–41 gestational weeks from all participants. Maternal blood samples and paired placental samples were collected from the third trimester. Placental samples were collected during the third trimester after cesarean section delivery. For the current study, 280 singleton pregnancies were selected including normal pregnancies (*n* = 188) and pregnancies with hypertensive disorders (*n* = 92) including EO-PE (*n* = 16), LO-PE (*n* = 47) and GH (*n* = 29). Normal controls included women without medical or obstetric complications. PE was defined as hypertension (systolic blood pressure ≥140 mmHg and/or diastolic blood pressure ≥90 mmHg, twice, 44 h apart) and proteinuria (≥0.3 g/day urine collection and/or ≥1+ on dipstick testing) after 20 weeks of gestation. PE was subclassified into early-onset PE (EO-PE) (onset before 34 weeks) and late-onset PE (LO-PE) (onset at or after 34 weeks). GH is a provisional diagnosis for women with new-onset hypertension without proteinuria after 20 weeks of gestation. Maternal, fetal, and infant records were retrospectively collected. All participants recruited in this study had no histories of chronic hypertension, diabetes mellitus, PE, liver disease, or chronic kidney disease.

### 3.2. DNA Preparation

Maternal blood (10 mL) was drawn and collected in EDTA tubes and immediately centrifuged at 1600× *g* for 10 min at 4 °C. Supernatant was re-centrifuged at 16,000× *g* for 10 min at 4 °C to minimize the additional maternal DNA and then aliquotted into 1 mL units for DNA extraction. DNA was extracted from placental tissues and maternal blood cells using the QIAamp DNA Mini kit (Qiagen, Hilden, Germany). Circulating cell-free DNA was extracted from 1 mL of maternal plasma using the QIAamp DSP Virus Kit (Qiagen) according to the manufacturer’s instructions.

### 3.3. Data Analysis of High-Resolution Tiling Arrays

To identify novel fetal-specific hypermethylated regions, we reanalyzed previously investigated tiling array data from blood samples from 2 non-pregnant normal women, and 4 pairs of fetal placentas and maternal blood samples from the first trimester. The normalized log_2_ value is the methylated DNA recovered by MBD capture: total input DNA signal ratio calculated by subtracting background for each probe, and represents a relative methylation level at each locus. We specified hypermethylation as a normalized log_2_ ratio > 0.5, hypomethylation as a normalized log_2_ ratio between 0.001 and 0.499, and unmethylation as a normalized log_2_ ratio = 0, as described previously [17].

### 3.4. Bisulfite Direct Sequencing

To confirm the MBD array data in the first trimester and validate the consistency of tissue-specific methylation levels, bisulfite direct sequencing was conducted in samples from the third trimester. Eight pairs of fetal placentas and maternal blood samples from normal pregnancies and 8 pairs of fetal placentas and maternal blood samples from pregnancies with PE were used. To further develop assays for detecting plasma circulating cell-free DNA, which are mainly short DNA fragments, we looked for loci of probe positions containing multiple differentially methylated CpG sites and additional flanked CpGs that are also differentially methylated within a short DNA stretch (approximately 250 bp). Both ENST00000433952:-7924 and ENST00000433952:-7988 regions were located nearby and therefore sequenced together, and ENST00000450830:-4439 regions were ruled out due to sequencing failure by numbers of poly T region in bisulfite converted sequences. Using an EpiTect Bisulfite Kit (Qiagen) DNA samples (1 μg) were bisulfite-converted, and then the bisulfite-converted DNA was amplified by PCR. The sequences of PCR primers are presented in Table S1. PCR products were purified using a PCR purification kit (Bioneer, Daejeon, Korea) and sequenced using a PRISM BigDye Terminator Cycle Sequencing Kit (Applied Biosystems, Foster City, CA, USA) according to the manufacturer’s instructions. Sequencing products were analyzed using a ABI 3130XL Genetic Analyzer (Applied Biosystems), and electropherogram traces were interpreted with DNA sequencing analysis software version 5.3 (Applied Biosystems).

### 3.5. Methylation Quantification of Bisulfite Direct Sequencing Data

The methylation ratio of each CpG site was calculated as the height of C peak/the height of (C + T) peak, as shown in the sequencing chromatogram extracted from the Chromas program (Version 2.32, Technelysium) [35]. A single C at the CpG site was considered to be completely methylated (100%), a single T was completely unmethylated (0%), and overlapping C and T was partially methylated (0%–100%).

### 3.6. Enrichment of Methylated DNA

We isolated the methylated cffDNA from circulating cell-free DNA extracted from maternal plasma using the MethylMiner^TM^ methylated DNA enrichment kit (Invitrogen, Carlsbad, CA, USA) according to the manufacturer’s instructions. MBD-biotin protein (3.5 μg) was conjugated to 10 μL Dynabeads M-280 Streptavidin for 1 h at room temperature. The conjugates were washed three times and resuspended in 1 volume of 1X Bind/Wash buffer. The capture reaction was achieved by the addition of 40 μL extracted circulating DNA to the MBD-magnetic beads on a rotating mixer for 24 h at 4 °C. The beads were then washed three times with 1X Bind/Wash buffer and the bound methylated DNA was eluted in a step-wise elution using increasing NaCl concentration in the elution buffer (600, 1000 and 2000 mM NaCl). Subsequently, eluted methylated DNA was concentrated and then eluted in a final volume of 30 μL using a DNA concentrator (Zymo Research Corp., Irvine, CA, USA). Validation was done using the control DNA (methylated DNA and unmethylated DNA) included in the kit according to the manufacturer’s recommendations.

### 3.7. Quantitative Real-Time PCR

Quantifications of methylated *DSCR3* and *RASSF1A* genes from circulating cell-free DNA in maternal plasma were performed in a duplex reaction. In addition, *SRY* and *GAPDH* concentrations were also quantified in circulating cell-free DNA extracted from 54 plasma samples in 92 pregnancies carrying a male fetus. Quantitative real-time PCR was performed using the ABI 7500 Real Time System (Applied Biosystems, Branchburg, NJ, USA). The reactions of the duplex PCR were performed in a volume of 20 μL, using 5 μL 4X NEXpro™ Dia PCR Master Mix (Geneslabs, Seongnam, Korea) and 6 μL methylated plasma DNA captured by MBD. Primers and probes were used at final concentrations of 250 nM each for *DSCR3*, *RASSF1A*, *SRY* and *GAPDH*. Sequences of primers, probes, and amplicons are shown in Table S1. Single-stranded synthetic DNA oligonucleotides specific to the *DSCR3* and *RASSF1A* amplicons (Bioneer, Daejeon, Korea) were serial diluted and employed as a standard curve. A standard curve of *SRY* or *GAPDH* was made by amplification of commercial male genomic DNA (Promega, Madison, WI, USA) at serial 10-fold dilutions. Each standard was amplified in triplicate and was included on every PCR plate. The concentrations of factors were expressed as copies/mL, and the standard factor of 6.6 pg was used to convert the data to copy numbers, as described previously [36]. Samples were amplified in triplicates and the average data reflected as the final results. According to the minimum information for publication of quantitative real-time PCR experiments (MIQE) guidelines [37], real-time PCR was highly sensitive enabling detection of more than 3 copies of the target.

### 3.8. Statistical Analysis

Comparisons between outcome groups were made by chi-square test or Fisher’s exact test for categorical variables and by the Mann-Whitney *U* test with post-hoc Bonferroni correction for continuous variables. We used locally weighted (Loess) regression to estimate a smooth, best fitting trend line. The Kruskal–Wallis test was also used to compare quantitative variables across multiple groups with subsequent pairwise analysis. Spearman’s rank correlation was used to calculate correlation coefficients. All data were analyzed using the SPSS software 12.0, and statistical significance was assigned at *p* < 0.05.

## 4. Conclusions

In conclusion, we identified a potential hypermethylated cffDNA epigenetic marker and demonstrated significantly increased concentrations of cffDNA (*DSCR3* and *RASSF1A*) in EO-PE before the onset of symptoms. Our findings suggest that the hypermethylated *DSCR3* gene could be a useful epigenetic marker for the prediction of EO-PE. However, this study is limited by its relatively small sample size and only included Korean women, and therefore further studies are warranted in larger cohorts to confirm the effectiveness of this marker.

## Supplementary Materials

Supplementary materials can be found at http://www.mdpi.com/1422-0067/16/12/26201/s1.
