# Supplementary material for: Quantification and Application of Potential Epigenetic Markers in Maternal Plasma of Pregnancies with Hypertensive Disorders
Source: Int J Mol Sci. 2015 Dec 15;16(12):29875–88. doi: 10.3390/ijms161226201 (PMC4691144; doi:10.3390/ijms161226201)
Supplement: Supplementary file 1 [file ijms-16-26201-s001.pdf]

Supplementary Materials: Quantification and Application of Potential Epigenetic Markers in Maternal Plasma of Pregnancies with Hypertensive Disorders

Hyun Jin Kim, Shin Young Kim, Ji Hyae Lim, Dong Wook Kwak, So Yeon Park and Hyun Mee Ryu

Table S1. Sequences of primers and probes according to assay.

**Assay**	**Target Regions [GenBank ID]**		**Sequences**
Bisulfite sequencing	*SOD1* [NG_008689.1]	Forward	5′- GTAATTTGTTTGTTTTTTTTTTTGTG -3′
		Reverse	5′- CAACAACATCTTATATACAAAAACC -3′
	DSCR3 [NG_009410.1]	Forward	5′- GTAAATATATGTAAAAATAGGAAGTG -3′
		Reverse	5′- CTTTTACTACATATAACTACACCCCC -3′
	C2CD2 [AP001745.1]	Forward	5′- GAGGACGATAAAAGGAATTAGTTTTT -3′
		Reverse	5′- ATTTTTATCTTATTTTTCTCAATTACAC -3′
	UMODL1 [AP001745.1]	Forward	5′- GATTTTTTTGGAGGAATTTTTTTTT -3′
		Reverse	5′- CAAAAATAAATAATTTACTCTACTCC -3′
	ENST00000433952:-7924—7988 [AL163278.2]	Forward	5′- GAATTGTTTAAGAGGTTTAAGTTTGG -3′
		Reverse	5′- CCTATAATTAAATTACATATAAAACAACC -3′
	RASSF1A [NG_023270.1]	Forward	5′- TAGTTTGGATTTTGGGGGAGG -3′
		Reverse	5′- CCCCAAATAAAATCGCCAC -3′
Real-time PCR	*DSCR3*	Forward	5′- CGTAGCGGCTTCTCGTG -3′
		Reverse	5′- GTTAGCCATCGGCTAGGTGG -3′
		Probe	5′- FAM-CCGAAGCGTCTGGCCTGTGTGCTCT-BHQ1 -3′
		Amplicon (125 bp)	5′- CGTAGCGGCTTCTCGTGGGCGAGTCCCTGTTCGCAGGTGACG TGTGGACCACGCTCTTCCGAAGCGTCTGGCCTGTGTGCTCTC GGGGAGGGGACGCAGGTCAGCCCACCTAGCCGATGGCTAA C -3′
	RASSF1A	Forward	5′- GAGCCTGAGCTCATTGAGCTG -3′
		Reverse	5′- ACCAGCTGCCGTGTGG -3′
		Probe	5′- HEX -CACCCGCTGGGCGCGC-BHQ1 -3′
		Amplicon (131 bp)	5′- GAGCCTGAGCTCATTGAGCTGCGGGAGCTGGCACCCGCTGG GCGCGCTGGGAAGGGCCGCACCCGGCTGGAGCGTGCCAAC GCGCTGCGCATCGCGCGGGGCACCGCGTGCAACCCCACACG GCAGCTGGT -3′
	SRY [NG_011751.1]	Forward	5′- AGATCAGCAGGGCAAGTAGT -3′
		Reverse	5′- TGAAACTTGCATTTCTCCGC -3′
		Probe	5′- FAM-CAGGGTACTAGGGGGTAGGCTGGTTG-BHQ1 -3′
	GAPDH [NG_007073.2]	Forward	5′- CCCCACACACATGCACTTACC -3′
		Reverse	5′- GTGGGAAGAGGGGAAGCTG -3′
		Probe	5′- HEX-AAAGAGCTAGGAAGGACAGGCAACTTGGC-BHQ1 -3′

A

Target sequences of *DSCR3* [NG_009410.1]

38629468 GCAAATATATGTAAAAATAGGAAGTGCGGTTTCCCAAAATGAGGTCTGTAAACAACTGATCTAGAAAATGTTCTGGAAAAAGTAAAAAAGGAT
CAGGATCTGAGGTCAACTGACCTCTCCCTGCGCTCTGGACAGGCAAACAGGCAAGGTTCCCTCTGAGGC**CG**TAG**CG**GCTTCT**CG**TGGG**CG**AGTCCC
TGTT**CG**CAGGTGA**CG**TGTGGACCA**CG**CTCTTC**CG**AAG**CG**TCTGGCCTGTGTGCTCT**CG**GGGAGGGGA**CG**CAGGTCAGCCCACCTAGC**CG**ATGGCT
AACAAGTCAGTTTGTTTTCTGAACGGAAGCTTAAACCTAGAAAAGTAACTGGGTTGGGGTGGGGGTGTAGCCACATGCAGTAAAAG **38629837**

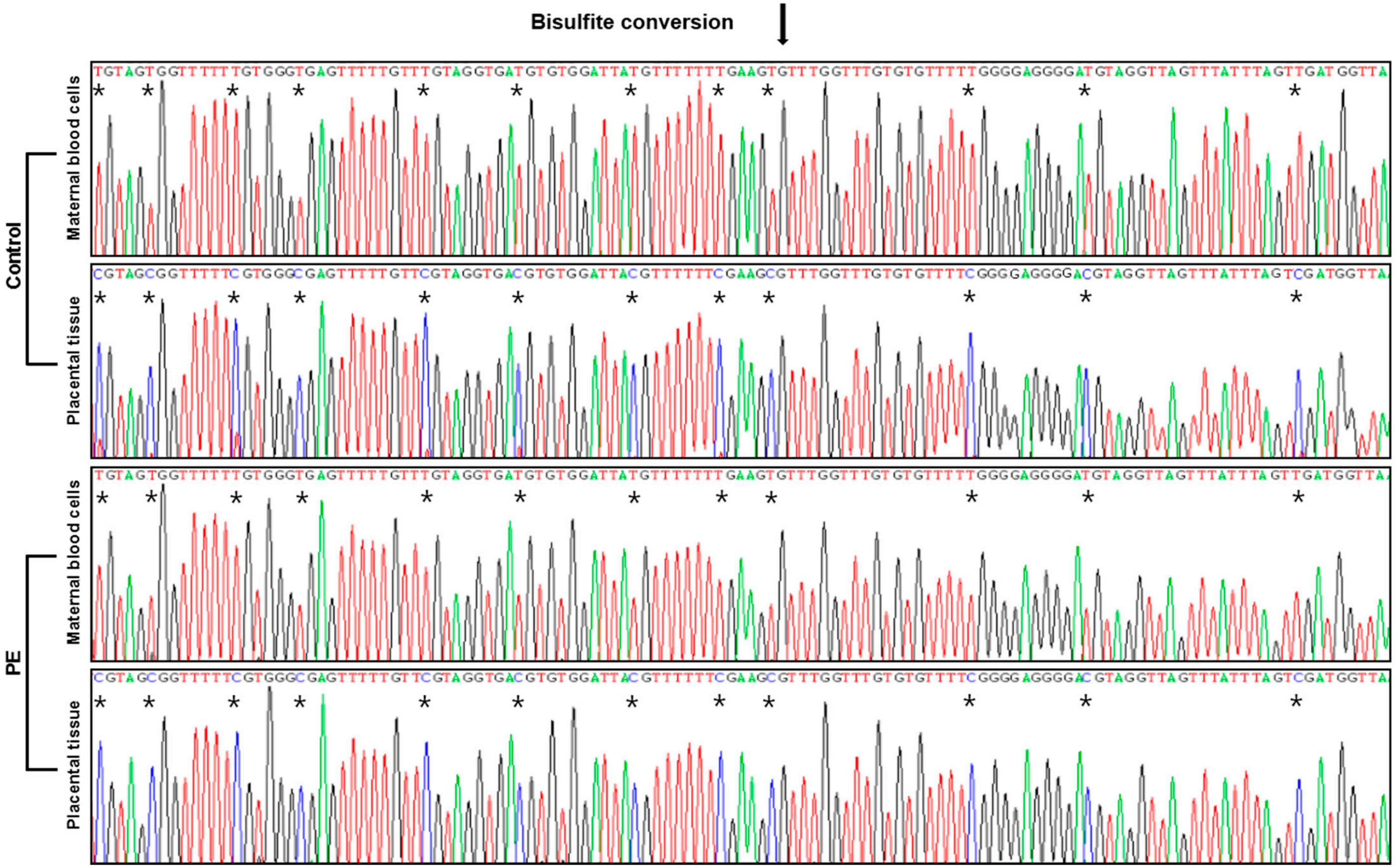

Figure S1. *Cont.*

B **Target sequences of *SOD1* [NG_008689.1]**

33037823 GTAACTTGCCTGCCTTTCTCCCTGTGTGGGACTCCTG**CG**GGTGAGAG**CG**TGGCTGAAGACAGC**CG**TGTTATGAAAGGGCCTCCTGTGCTGT**CG**AGGTTGTGCTCTGTGAATGTCAT CCCCTGGTGCACAGCAGCACCTTCTACACAGGATACAGTTGGAATGCCGCCCCCTCGAGTTGTGTAAGGCAGCAGCCTTGGCCCTTGCACATAAGATGCTGTTG **33038042**

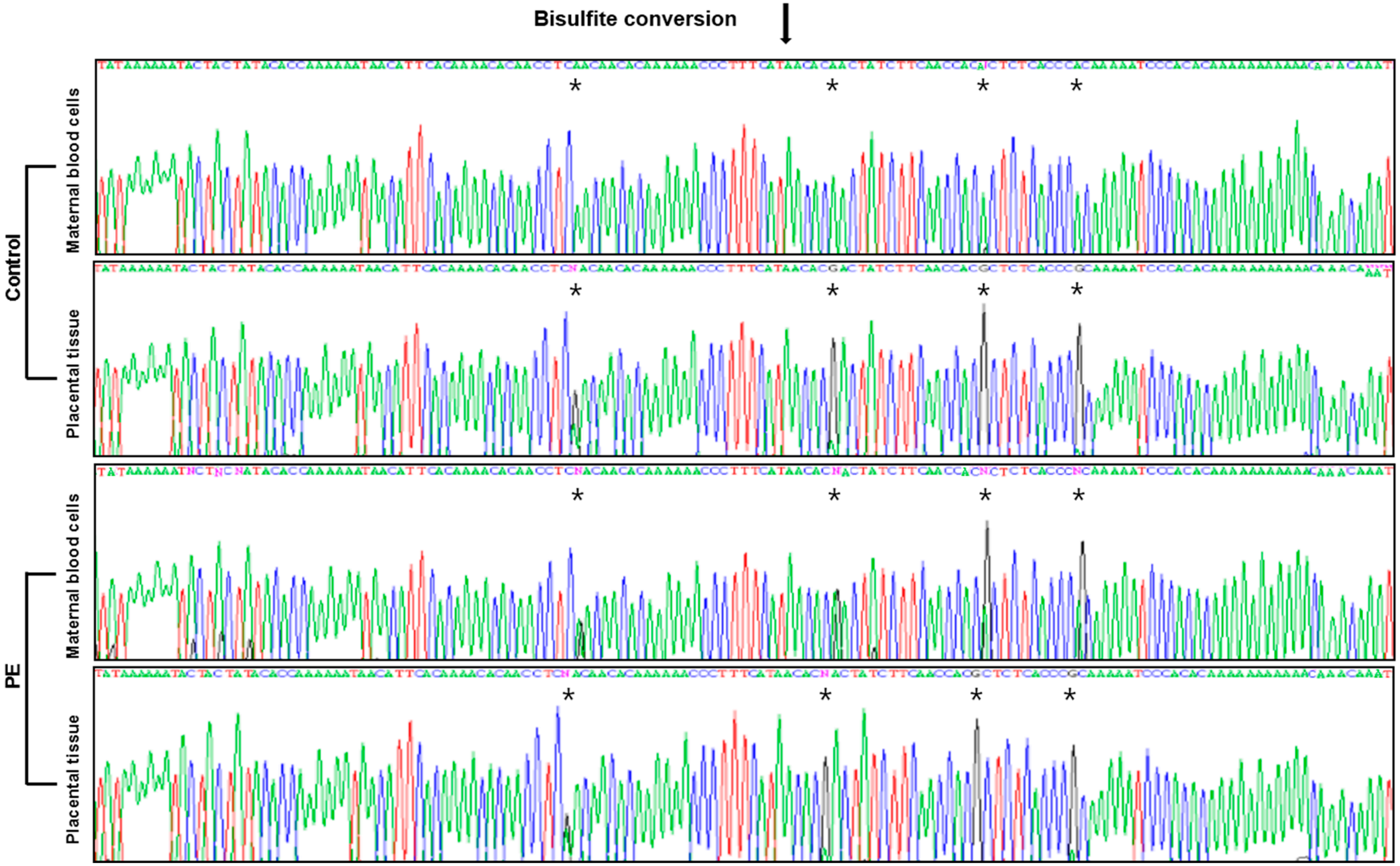

Figure S1. *Cont.*

C

Target sequences of *C2CD2* [AP001745.1]

43316042 GAGGACGACAAAAGGAACCAGCTTCTTCCTGTGGGTGTCACAG**CG**AGGT**CG**CCTGGCCACATCAGGTACCAGAG**CG**AG**CG**CCCTCACCT
GATAGGCCCTGTACAACCTCAGCCACAGCACTGTCAGGAGGAACA**CGCG**GAACTAGCAACCTAGGAGGGTAAAGG**CG**GAGTTGGGAGGGAACA**CG**
AGGCAGGCAGGT**CG**GCTGGCTGCTGAGCTACAGGCTGCACTCCTAGGA**CG**TCTA**CG**TGTAATTGAGAAAAATAAGACAAAAAT **43316308**

Bisulfite conversion

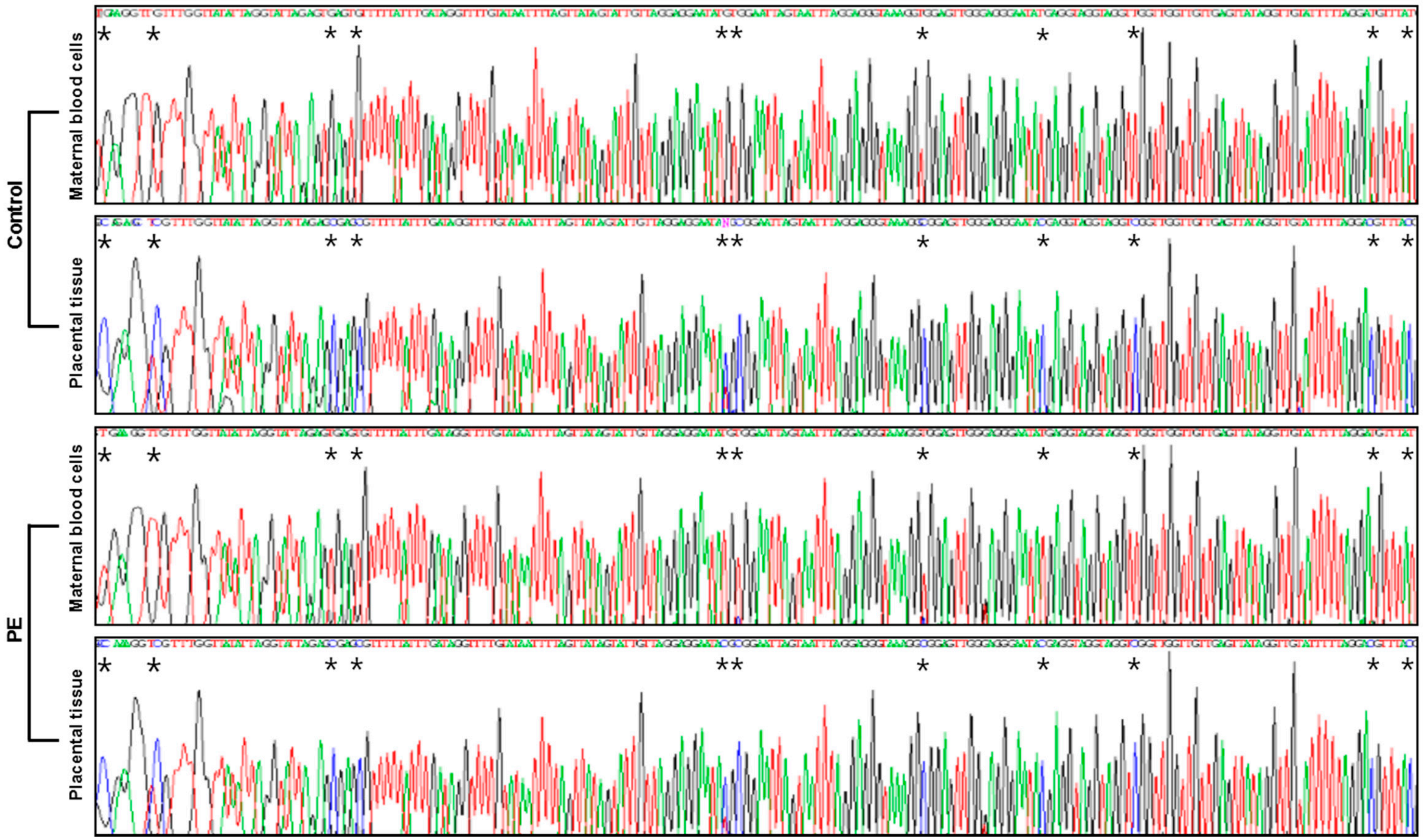

Figure S1. *Cont.*

D

Target sequences of *UMODL1* [AP001745.1]

43483745 GACCCCCTGGAGGAATCCCTCCCTCTAAGACTCTGGGACTGGTGCACGCAAGGAGCTATCGTGAACATTGCTCCCAACTGGC**CG**CTTGCT
TGTCCCC**CG**GCTCCCCTTGGCCCCAGTGG**CG**GCTTTGCCTGAATTAGAGGG**CG**TGAGAGCCACCTGTGTCTCAGCACTGCAATTAAAGCAGGAAGC
CCTTT**CG**GAAGCAGC**CG**TGTGCACCAGCCTCCCATGGGTGGAGCAGAGCAAACCACCCACTTCTGCCCTCTG **43484002**

Bisulfite conversion ↓

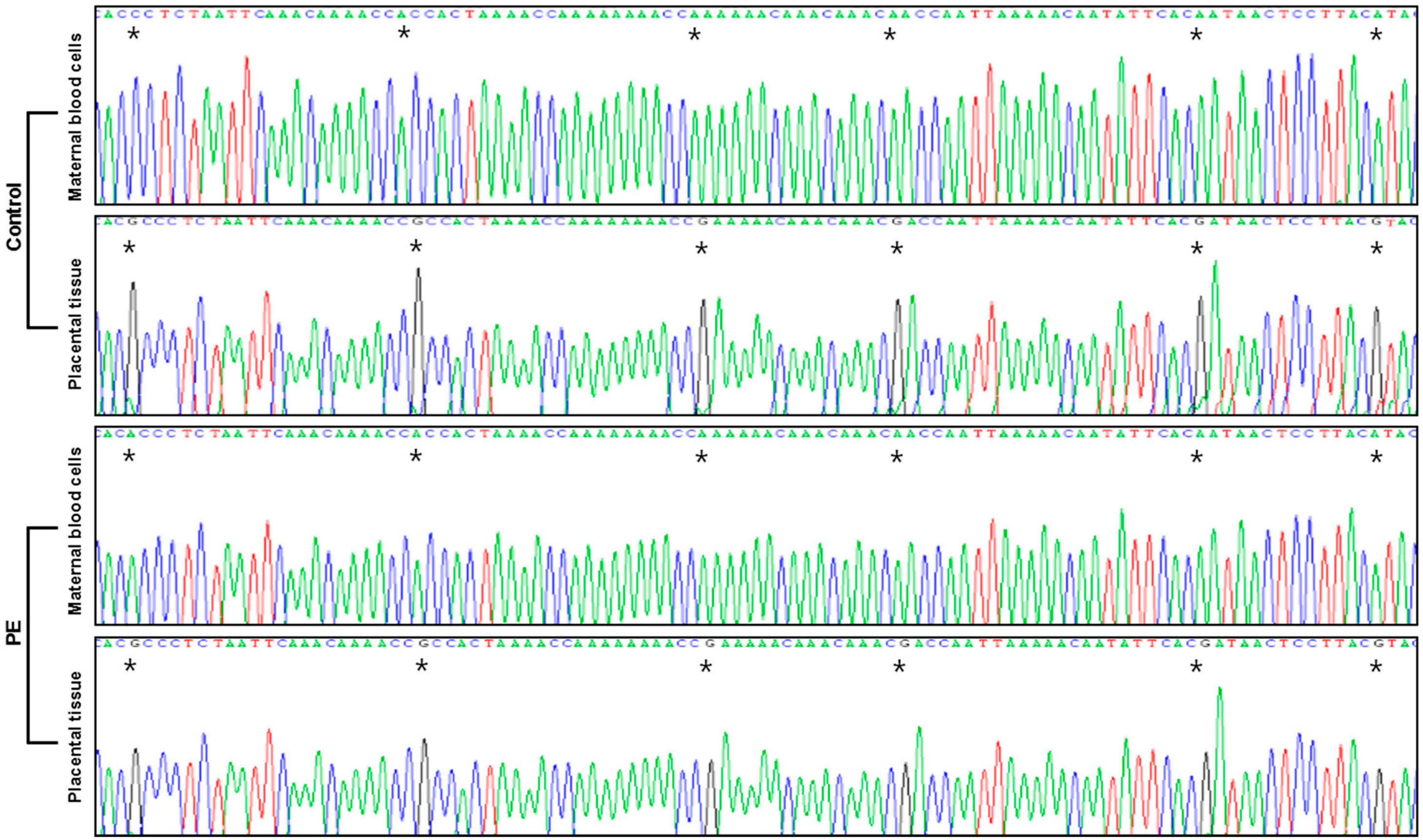

Figure S1. *Cont.*

E

Target sequences of ENST00000433952: -7924 ~ ENST00000433952: -7988 [AL163278.2]

40357575 GAACTGTTCAAGAGGCCCAAGCCTGGTGCTCAGAAACTTGGCAGGATCAAGCATCTCGCCCAGGAATTCATCCCCTGCTTGTCTAAGC**CG**G
CTGGCTCT**CG**TGACTGACT**CG**GAACAACAGAGCAGATGTTTG**CG**TGGGAGGCAAGCCTCACCCAACATCTGTCCTG**CG**G**CG**GGAAGGCCTGGGTGT
TCACAGATAGAGCTGGAGTTCCC**CG**GTGGGTGGCACAGACAATTAGCTGGGGCTGCCTCACATGTAATCTAATTACAGG **40357840**

Bisulfite conversion

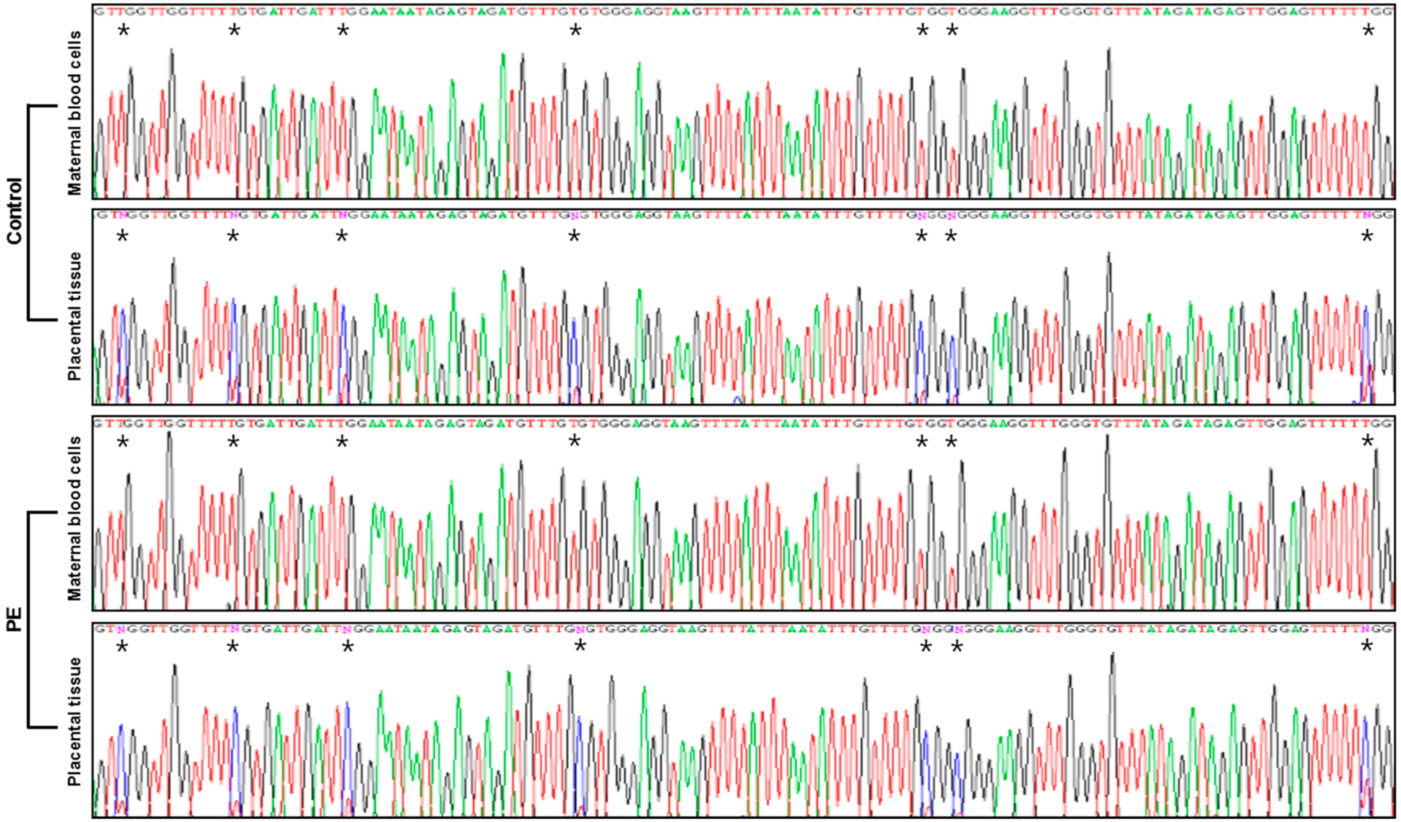

Figure S1. *Cont.*

F

Target sequences of *RASSF1A* [NG_023270.1]

50378016 CAGTCTGGATCCTGGGGGAGGCGCTGAAGTCGGGGCCCGCCCTGTGGCCCCGCCCGGCCCGCGCTTGCTAGCGCCCAAAGCCAGCGAA
GCACGGGCCCAACCGGGCCATGTCGGGGGAGCCTGAGCTCATTGAGCTG**CG**GGAGCTGGCACC**CG**CTGGG**CGCG**CTGGGAAGGGC**CG**CACC**CG**
GCTGGAG**CG**TGCCAA**CGCG**CTG**CG**CAT**CGCGCG**GGGCAC**CGCG**TGCAACCCCACA**CG**GCAGCTGGTCCCTGGCCGTGGCCACCGCTTCCAGCC
CGCGGGGCCCGCCACGCACACGTGGTGCGACCTCTGTGGCGACTTCATCTGGGG **50378343**

Bisulfite conversion

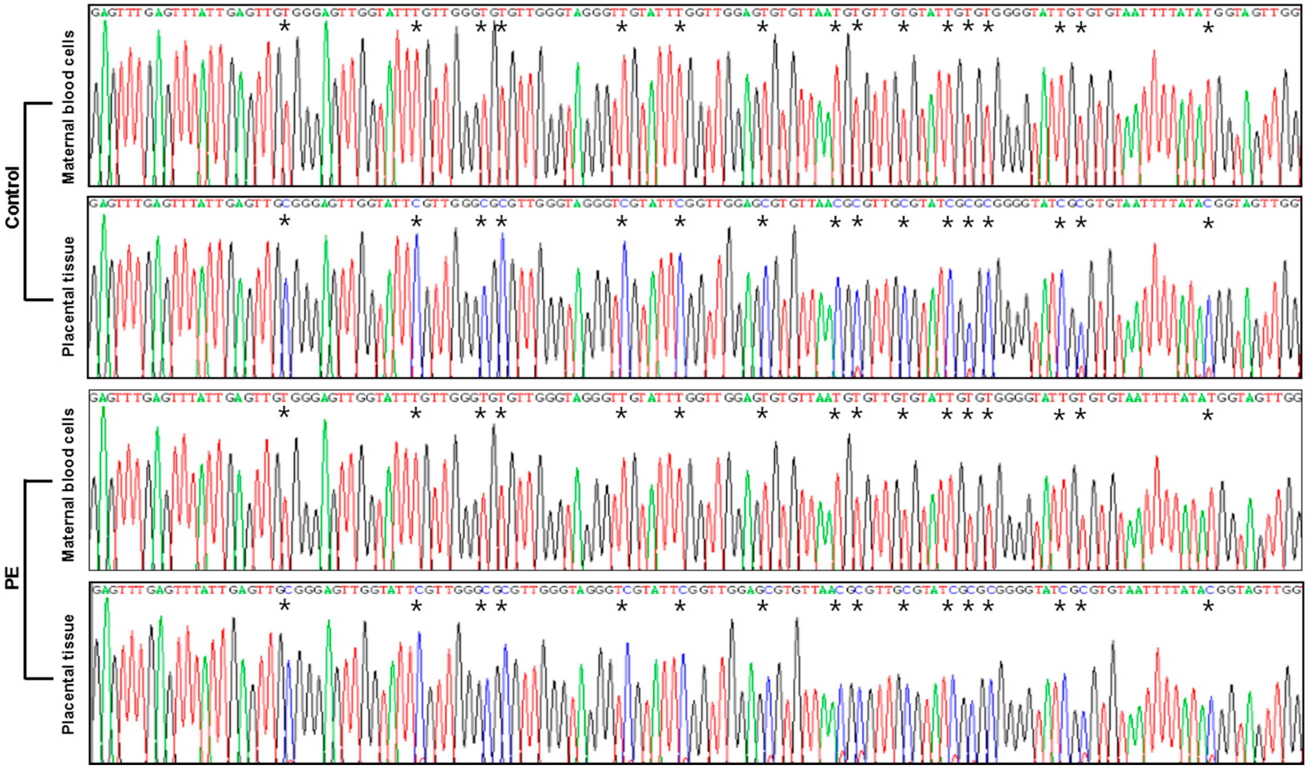

Figure S1. DNA methylation levels of each CpG site in 6 regions (**A**–**F**) by bisulfite direct sequencing. The asterisks represent CpG sites in each region. Red and blue peaks in sequences indicate T and C bases, respectively. Probe positions are underlined, and real time PCR regions are highlighted in yellow. The bold characters in the sequences indicate methylation CpG sites of the each region. *SOD1* and *UMODL1* sequences are written in the reverse orientation (3′ to 5′ direction).
